# Tissue‐specific targeting of the ARF6 signalling axis for type 2 diabetes and obesity: From mechanism to nanodelivery

**DOI:** 10.1002/ctm2.70692

**Published:** 2026-05-14

**Authors:** Yangyang Wang, Yan He, Zhiming Xiu, Huiyan Wang

**Affiliations:** ^1^ Jilin Collaborative Innovation Centre for Antibody Engineering Jilin Medical University Jilin China

**Keywords:** ADP‐ribosylation factor 6, glucose transporter type 4 translocation, insulin resistance, insulin secretion, nanodelivery, obesity‐associated inflammation, targeted therapy

## Abstract

Disorders of glucose metabolism, particularly type 2 diabetes and obesity, remain major therapeutic challenges because they involve dysfunction across multiple tissues, including pancreatic β‐cells, peripheral insulin‐sensitive tissues, and immune cells. ADP‐ribosylation factor 6 (ARF6) is a regulator of membrane trafficking and cytoskeletal dynamics and may represent a mechanistically relevant link across these compartments. In this review, we summarise evidence suggesting that ARF6 may contribute to sustained second‐phase insulin secretion, glucose transporter type 4 trafficking and recycling, and metabolic inflammation through effects on receptor and membrane trafficking. We also discuss pharmacological and nucleic acid‐based approaches targeting ARF6 or its regulatory network. Current evidence suggests that direct systemic inhibition may be difficult to translate because of off‐target risks, including possible disruption of endothelial barrier integrity. In addition, because cellular uptake of some delivery systems depends on endocytic pathways associated with ARF6, broad inhibition may also interfere with drug entry. Given these limitations, tissue‐targeted and microenvironment‐responsive nanodelivery systems may provide a more feasible strategy for modulating the ARF6 axis with greater spatial and temporal control. Overall, this review presents ARF6 as a potentially important mechanistic and translational entry point within the broader network that regulates glucose homeostasis, rather than as a single master regulator.

## Key points

- ARF6 acts as a cross‐tissue hub linking membrane trafficking to glucose metabolism.
- In β‐cells, ARF6 sustains second‐phase insulin secretion and release‐site reuse.
- ARF6 drives GLUT4 endocytic recycling to preserve peripheral insulin sensitivity.
- ARF6 amplifies obesity‐driven tissue inflammation via immune receptor recycling.
- Targeted nanodelivery overcomes systemic ARF6 toxicity and the uptake paradox.

## Graphical abstract

Multitissue targeting and nanodelivery strategies centred on ADP‐ribosylation factor 6 for type 2 diabetes and insulin resistance.

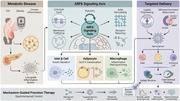

## 1 INTRODUCTION

**FIGURE 1:**
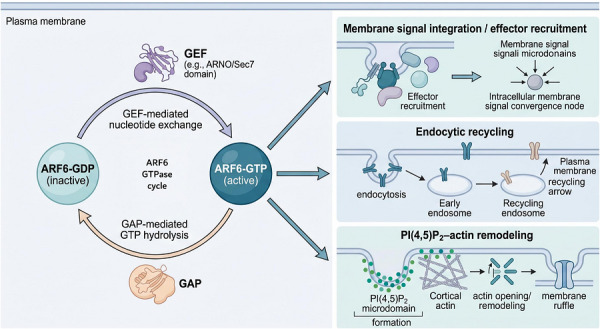
Overview schematic of the ADP‐ribosylation factor 6 (ARF6) signalling axis in metabolic regulation. This conceptual overview illustrates the canonical ARF6 GDP/GTP cycle and its principal downstream functional outputs. In the left module, guanine nucleotide exchange factor (GEF)‐mediated nucleotide exchange converts inactive ARF6‐GDP into active ARF6‐GTP, whereas GTPase‐activating protein (GAP)‐mediated GTP hydrolysis returns ARF6 to the GDP‐bound state. In the right modules, activated ARF6‐GTP is shown to support three major membrane‐associated functions relevant to metabolic regulation: effector recruitment and membrane signal integration, endocytic recycling, and PI(4,5)P_2_‐associated cortical actin remodelling. These downstream outputs provide the conceptual basis for the later discussion of ARF6 in β‐cell insulin secretion, peripheral glucose transporter type 4 (GLUT4) trafficking, and inflammatory receptor turnover. Arrows extending from ARF6‐GTP indicate functional outputs of the activated state rather than additional steps within the nucleotide cycle. Key mechanistic nodes highlighted include PI(4,5)P_2_ microdomain formation, cortical actin remodelling, early/recycling endosomes, and membrane‐associated signal integration.

Glucose metabolism disorders, especially type 2 diabetes mellitus and obesity‐related insulin resistance, remain major challenges to metabolic health. These conditions increase the risk of cardiovascular disease and other systemic complications. Their development involves several interconnected defects, including reduced insulin secretion by pancreatic β cells, impaired insulin sensitivity in peripheral tissues, and persistent low‐grade inflammation driven by immune‐metabolic imbalance.^1^ Current therapies often improve only part of this process. In many cases, they do not adequately address all three features at the same time. This gap highlights the need to identify regulatory pathways that may connect insulin secretion, insulin sensitivity, and inflammation. ADP‐ribosylation factor 6 (ARF6), a small guanosine triphosphatase (GTPase), may represent one such pathway (Figure 1). ARF6 is a membrane‐associated GTPase that regulates membrane trafficking, phospholipid organisation, and cortical cytoskeletal remodelling. Because these processes are important in several metabolically active cell types, ARF6 may influence glucose homeostasis at multiple levels. As outlined in Figure 1, the ARF6 signalling axis can be viewed as a membrane‐centred network linking three major outputs: insulin secretion in β cells, glucose transporter type 4 (GLUT4) trafficking in peripheral tissues, and inflammatory receptor recycling in immune cells. This framework helps organise the review, moving from core ARF6 biology to tissue‐specific dysfunction and then to possible intervention strategies. ARF6 cycles between an active guanosine triphosphate‐bound state and an inactive guanosine diphosphate‐bound state. Through this cycle, it regulates membrane phospholipid remodelling, vesicle trafficking, and actin dynamics.^2^ Guanine nucleotide exchange factors (GEFs) activate ARF6 by promoting nucleotide exchange, whereas GTPase‐activating proteins (GAPs) terminate signalling by accelerating GTP hydrolysis. In this way, ARF6 is linked to membrane signal integration, endocytic recycling, and phosphatidylinositol 4,5‐bisphosphate [PI(4,5)P_2_]‐associated actin remodelling. These functions suggest that ARF6 could affect several aspects of metabolic regulation. In β cells, it may help maintain insulin granule trafficking and replenishment.^3^ In adipocytes and muscle, it may support GLUT4 recycling and sustained glucose uptake.^4^ In immune cells, it may also contribute to chronic inflammation by regulating receptor trafficking and related membrane‐dependent processes.^5^ At the same time, ARF6 is unlikely to be the only critical regulator of glucose metabolism. Other signalling pathways also contribute to β‐cell function, insulin responsiveness, and inflammatory control. For that reason, ARF6 is better viewed here as a promising regulatory node rather than a single master switch. Its potential importance lies in the possibility that one membrane‐trafficking pathway may influence several disease‐relevant processes across tissues.

Therapeutic modulation of ARF6 also remains challenging. ARF6 participates in essential housekeeping functions, including maintenance of cell architecture and endothelial barrier integrity. As a result, systemic inhibition may cause substantial off‐target effects.^6^ This concern suggests that spatiotemporal control will likely be necessary for any future ARF6‐directed strategy. Recent progress in nanomedicine, such as tissue‐targeting ligands and microenvironment‐responsive carriers, may offer a practical way to concentrate ARF6 modulators at disease sites while limiting unwanted effects in healthy tissues.^7^ This review summarises the current evidence linking ARF6 to glucose metabolism disorders and discusses how targeted nanodelivery may help translate ARF6‐directed modulation into a more feasible therapeutic approach.

## 2 CORE BIOLOGY OF THE ARF6 SIGNALING AXIS IN GLUCOSE METABOLISM

### 2.1 The ARF6 GDP/GTP cycle and membrane‐trafficking logic

**FIGURE 2:**
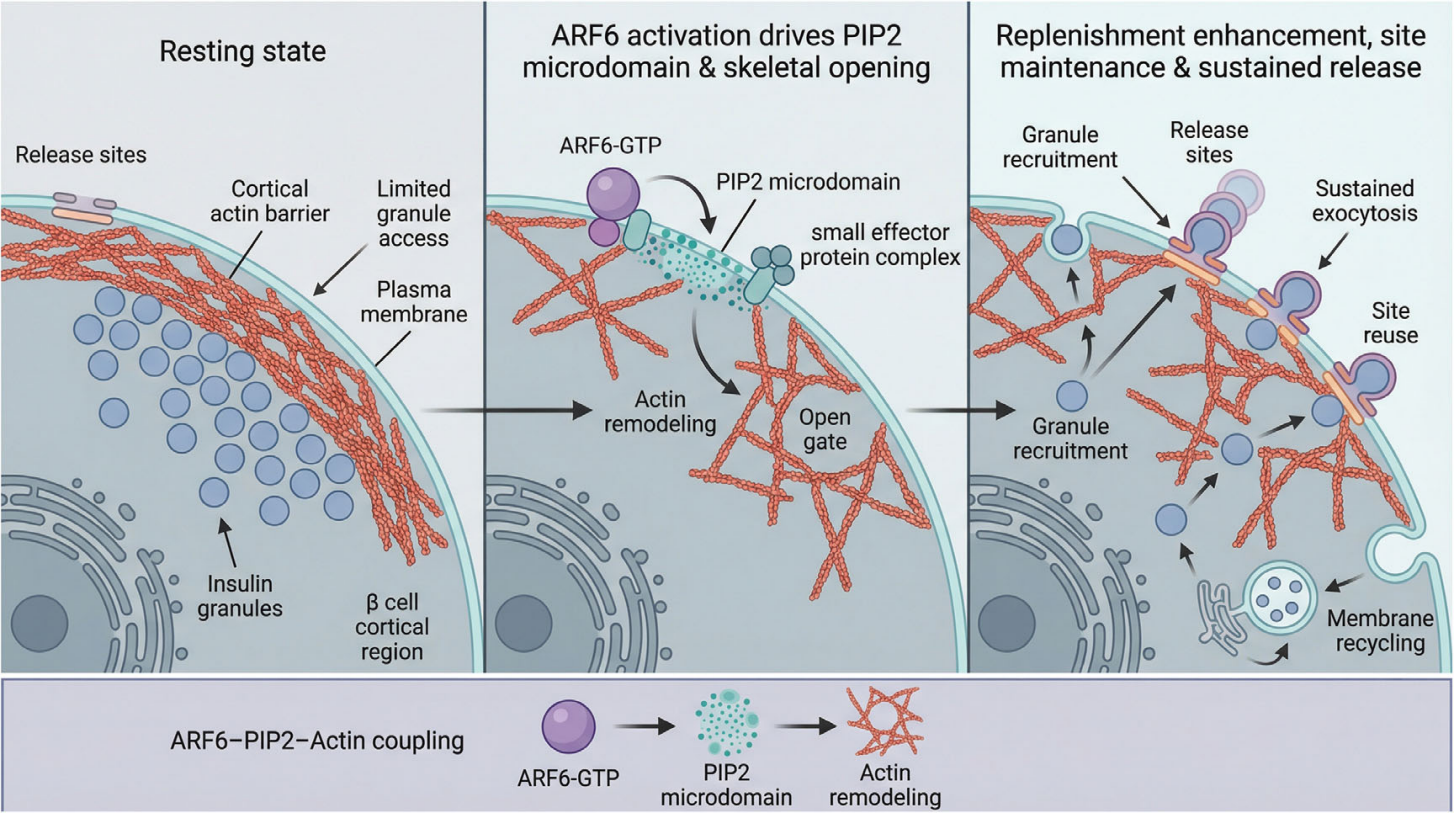
Working model of ARF6–PI(4,5)P_2_–actin coupling at the β‐cell cortex. This figure illustrates three functional states. Left: In the resting state, dense cortical actin restricts access of insulin granules to release sites. Middle: ADP‐ribosylation factor 6 (ARF6) activation at the plasma membrane promotes PI(4,5)P_2_ microdomain formation and localised actin remodelling, thereby creating permissive openings in the cortical barrier. Right: These changes enhance granule recruitment, maintain functional release sites, support repeated exocytosis, and facilitate membrane recycling. The schematic summarises the mechanistic coupling proposed to underlie second‐phase insulin secretion. Key nodes highlighted include ARF6‐GTP, PI(4,5)P_2_‐enriched membrane domains, cortical actin remodelling, and secretion‐site reuse.

ARF6 functions as a molecular switch. Its activation is driven by GEFs, such as ADP‐ribosylation factor nucleotide‐binding site opener (ARNO), whereas its inactivation is mediated by GAPs.^8^ In metabolic settings, activated ARF6 appears to coordinate two closely linked processes: membrane phospholipid remodelling and cortical actin reorganisation (Figure 2). Figure 2 summarises this coupling framework. It shows how ARF6‐dependent phosphatidylinositol 4,5‐bisphosphate [PI(4,5)P_2_] microdomain formation and local actin rearrangement may convert a relatively restrictive cortical region into a membrane area more permissive for vesicle trafficking. This model suggests that the biological importance of ARF6 does not lie simply in whether it is activated, but in how it coordinates lipid remodelling, actin dynamics, and trafficking output in a spatially controlled manner. Mechanistically, ARF6 can recruit lipid kinases that promote PI(4,5)P_2_ production, thereby helping generate local membrane microdomains required for vesicle docking and fusion.^9^ At the same time, ARF6 may support local remodelling of the cortical actin network, which can transiently reduce the physical barrier that limits vesicle access to the plasma membrane.^10^ ARF6 also contributes to the endocytic recycling of plasma membrane proteins and may influence the surface abundance of receptors and transporters.^11^ These functions—lipid organisation, actin remodelling, and membrane recycling—may provide a mechanistic basis for the three disease‐relevant contexts discussed in this review: sustained insulin secretion, GLUT4 trafficking, and inflammatory receptor turnover. Taken together, current evidence supports the view that ARF6 is an important membrane‐trafficking regulator. In the context of this review, its relevance lies in its potential to couple nucleotide‐state cycling with receptor/transporter recycling and actin‐dependent membrane remodelling.

### 2.2 ARF6‐dependent membrane phospholipid remodelling and cortical actin coupling

Genetic perturbation studies further support the biological relevance of the ARF6 pathway. Loss‐of‐function models and cell type‐specific manipulations suggest that disruption of this axis can alter membrane trafficking, cytoskeletal remodelling, and downstream metabolic phenotypes in a context‐dependent manner. At the same time, ARF6 is unlikely to act in isolation. Its contribution may be shaped by broader regulatory layers, including transcriptional, post‐transcriptional, and epigenetic control of ARF6 itself or of its regulatory partners. These factors may influence how strongly the pathway contributes across tissues and disease settings. Because this review focuses on mechanism‐to‐translation links, these regulatory layers are not discussed in detail here. However, they remain important topics for future target validation and for the development of more tissue‐selective therapeutic strategies. This coupling framework may be especially relevant in β‐cell physiology. Sustained secretion depends on repeated granule access to the plasma membrane and on preservation of functional release sites. ARF6‐dependent control of membrane organisation and cortical actin remodelling could contribute to these processes, although the extent of this contribution likely varies with experimental context.

## 3 ARF6 SUSTAINS SECOND‐PHASE INSULIN SECRETION IN β‐CELLS

### 3.1 Why ARF6 is positioned in the sustained second phase of glucose‐stimulated insulin secretion

**FIGURE 3:**
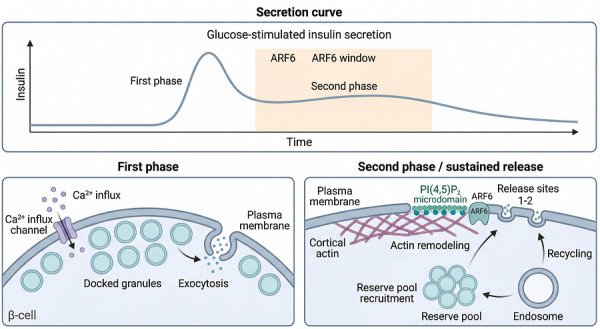
ADP‐ribosylation factor 6 (ARF6)‐associated control of biphasic glucose‐stimulated insulin secretion. The top panel shows the classical biphasic insulin secretion curve after glucose stimulation, with the proposed “ARF6 window” superimposed over the sustained second phase. The lower left panel depicts the first phase, which is dominated by Ca^2+^‐triggered exocytosis of predocked granules. The lower right panel illustrates the second phase, during which ARF6 is proposed to support PI(4,5)P_2_ organisation, cortical actin remodelling, granule recruitment from reserve pools, release‐site maintenance, and endosomal recycling. This schematic emphasises that ARF6 primarily contributes to sustained secretion rather than initial triggering.

Glucose‐stimulated insulin secretion (GSIS) includes a sustained second phase that helps limit postprandial increases in blood glucose. This phase depends on continuous recruitment of reserve granules to the plasma membrane and efficient reuse of exocytic sites (Figure 3).^12^ Figure 3 relates the classical biphasic GSIS curve to the cellular events discussed in this section. The upper panel indicates that the proposed window of ARF6 activity may overlap more with the sustained second phase than with the rapid first‐phase burst. The lower panel contrasts the Ca^2+^‐triggered release of predocked granules with processes that may depend more strongly on ARF6, including granule replenishment, actin remodelling, and membrane recycling. This framework suggests that ARF6 may act at the level of sustained secretory capacity rather than acute triggering alone. ARF6 is discussed here as a target‐relevant node because second‐phase GSIS depends not only on stimulus‐induced exocytosis, but also on continued granule recruitment, maintenance of functional release sites, and membrane recycling. These processes are likely constrained by membrane trafficking and cortical actin remodelling. Consistent with this view, ARF6 has increasingly been implicated as a contributor to sustained secretory output.^13^

### 3.2 ARF6 supports secretion‐site maintenance through PI(4,5)P_2_ homeostasis

Simultaneously, ARF6‐mediated PI(4,5)P_2_ generation stabilises secretion hotspots, ensuring that SNARE fusion complexes can be repeatedly assembled and utilised.^14^ Consequently, ARF6 deficiency typically spares initial acute insulin release but triggers a rapid collapse of the sustained second‐phase plateau.

### 3.3 ARF6‐dependent actin remodelling enables granule mobilisation and replenishment

During high‐glucose stimulation, ARF6 may locally remodel the dense cortical actin network beneath the β‐cell membrane. This remodelling could create a more permissive path for reserve granules to move toward the cell surface.^13^, ^15^ ARF6 may also couple exocytosis to compensatory endocytic retrieval during repeated stimulation. This process could limit excessive membrane expansion and support recycling of key exocytic components back into the releasable pool.^3^ This mechanistic profile helps explain why β‐cell‐directed delivery strategies discussed later in the review are framed around preserving sustained secretion capacity rather than simply amplifying acute secretory triggering.

## 4 ARF6 DRIVES GLUT4 TRANSLOCATION AND RECYCLING IN PERIPHERAL TISSUES

### 4.1 ARF6 in the trafficking execution layer of insulin sensitivity

**FIGURE 4:**
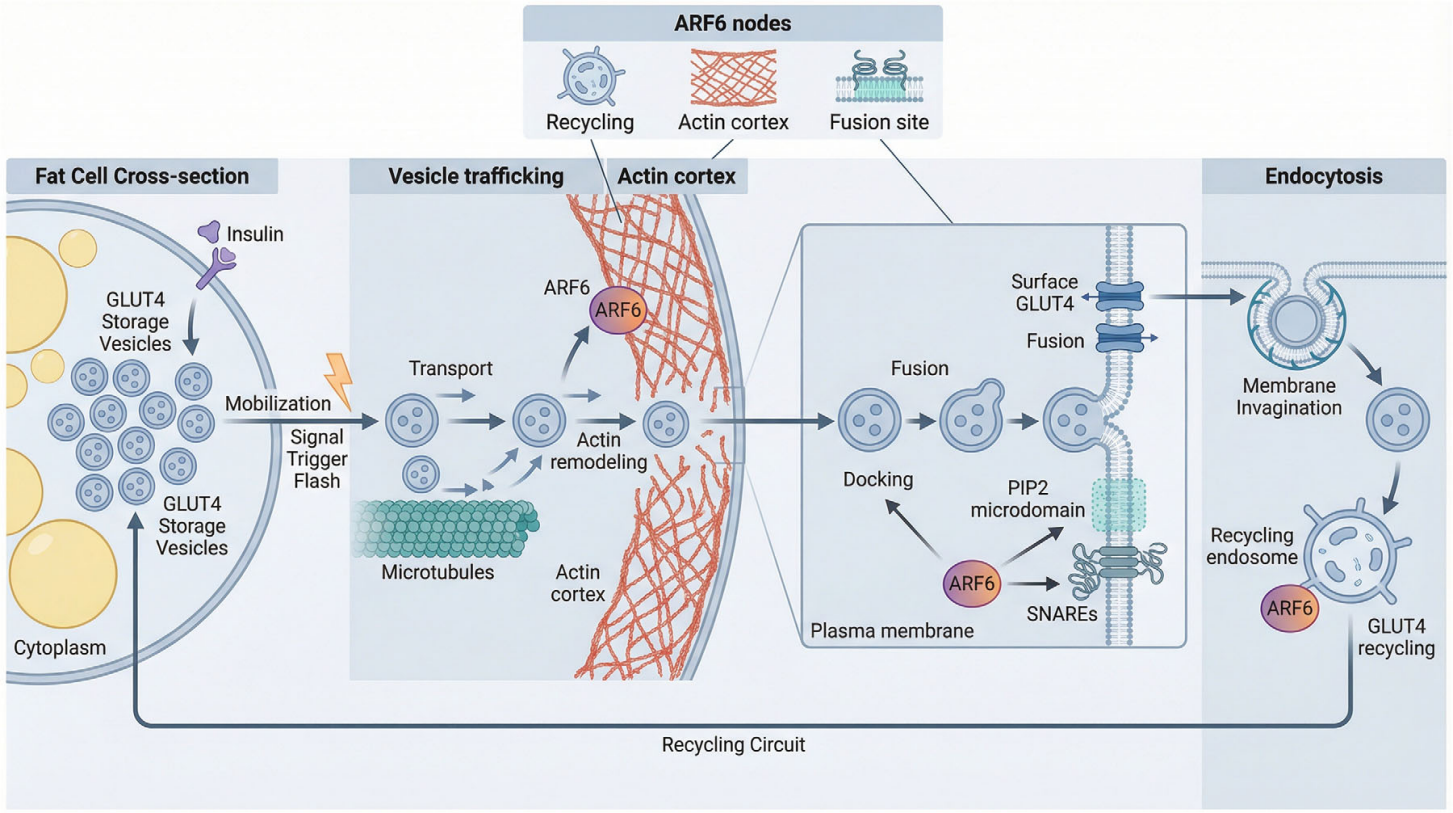
ADP‐ribosylation factor 6 (ARF6) regulatory nodes in glucose transporter type 4 (GLUT4) translocation and recycling. This schematic depicts the trafficking route of GLUT4 vesicles in insulin‐responsive cells. Insulin signalling mobilises GLUT4 storage vesicles, which are transported toward the cell periphery and must traverse the actin cortex before docking and fusing with the plasma membrane. ARF6 is proposed to regulate localised actin remodelling, PI(4,5)P_2_‐enriched fusion sites, and post‐endocytic sorting into recycling endosomes, thereby influencing both acute surface delivery and long‐term maintenance of the GLUT4 reservoir. The figure highlights how impaired recycling can contribute to insulin resistance even when proximal signalling remains partially intact. Key trafficking modules include GLUT4 storage vesicles, actin‐gated membrane access, plasma membrane fusion, endocytic retrieval, and recycling endosomes.

Peripheral insulin sensitivity depends largely on efficient GLUT4 translocation to the plasma membrane in adipocytes and skeletal muscle. In insulin‐resistant states, glucose uptake may remain impaired even when canonical PI3K‐Akt signalling is only partly disrupted. This pattern suggests that defects can arise not only from upstream signalling failure, but also from abnormalities in the vesicular trafficking machinery that executes transporter delivery.^16^ ARF6 is of interest in this context because impaired insulin sensitivity may reflect defects in post‐signalling trafficking steps. These include endocytic sorting, recycling efficiency, and reinsertion of GLUT4 into the plasma membrane. ARF6 has been proposed as a regulator of key post‐endocytic sorting decisions that influence GLUT4 availability (Figure 4).^8^ Figure 4 summarises the trafficking itinerary relevant to insulin‐responsive GLUT4 handling. This includes mobilisation of storage vesicles, cortical actin remodelling, docking and fusion at the plasma membrane, and subsequent endocytic recycling. In this framework, ARF6 may act at multiple points in the GLUT4 pathway rather than at a single terminal fusion step. This view also suggests that reduced insulin sensitivity can arise from defects in trafficking execution, even when upstream signalling is only partially impaired.

### 4.2 ARF6‐associated endocytic sorting and recycling of GLUT4

After endocytosis, ARF6 may support endosomal membrane tubulation and vesicle budding, thereby facilitating the return of GLUT4 to a mobilisable intracellular storage pool.^17^ When ARF6‐dependent recycling is impaired, more GLUT4 may be retained in endosomal compartments or redirected toward lysosomal degradation. This change could gradually reduce the intracellular reservoir available for insulin‐responsive mobilisation.^18^ From this perspective, ARF6 may influence both the recycling flux and the spatial availability of GLUT4. This provides a possible trafficking‐based explanation for impaired peripheral glucose uptake that is not limited to defects in upstream kinase signalling. Accordingly, Figure 4 presents insulin resistance as a disorder of ongoing GLUT4 cycling rather than only a defect in proximal insulin signalling. This trafficking‐centred interpretation also helps explain the rationale for the adipose‐targeted strategies discussed later in this review. These approaches are considered as potential ways to improve local GLUT4 recycling efficiency, rather than simply further increasing upstream signalling activity.

## 5 ARF6 AMPLIFIES OBESITY‐ASSOCIATED INFLAMMATION AND MACROPHAGE INFILTRATION

### 5.1 ARF6 as an amplifier of the metabolic inflammatory microenvironment

**FIGURE 5:**
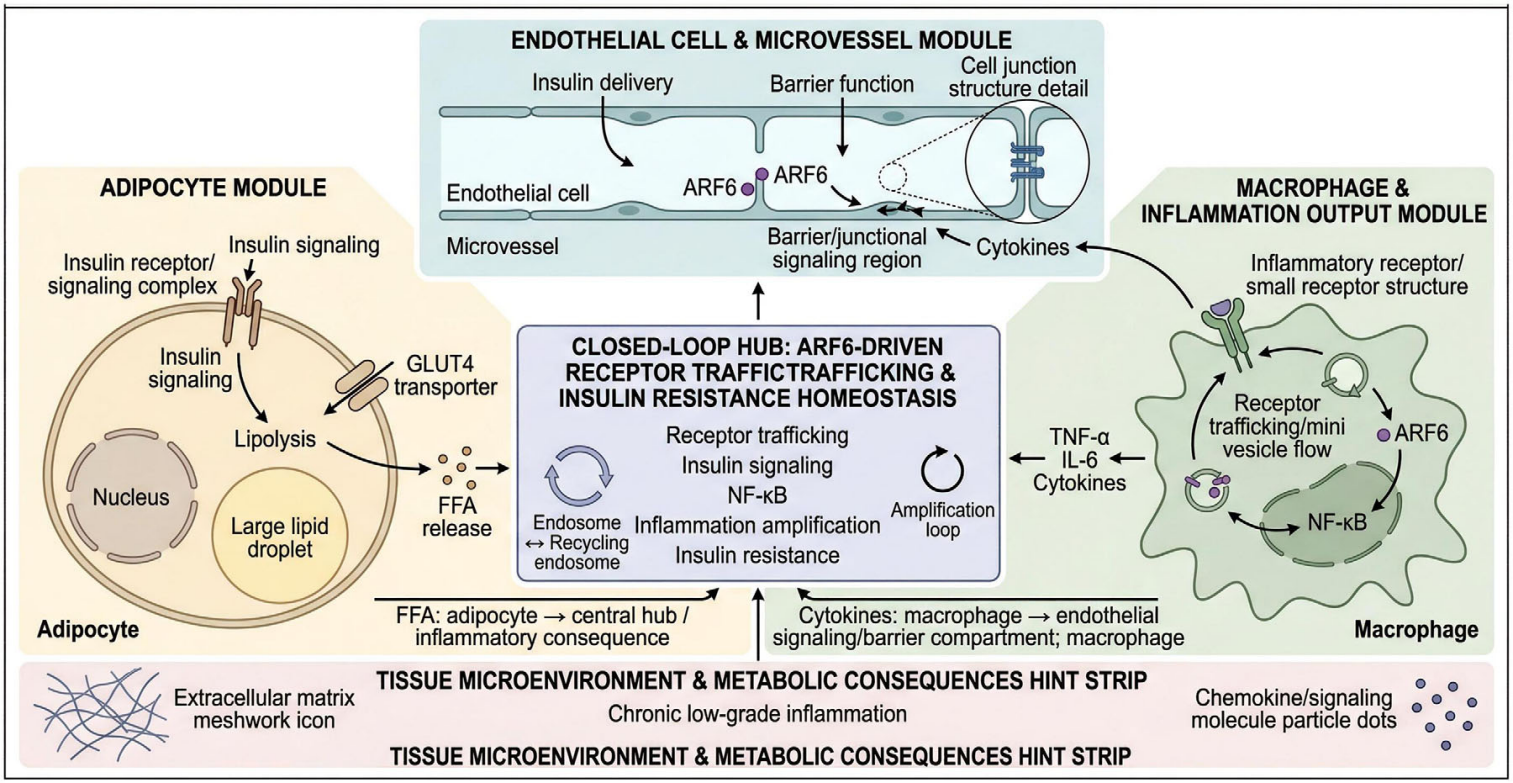
Closed‐loop model of ADP‐ribosylation factor 6 (ARF6)‐dependent inflammatory amplification in the metabolic tissue microenvironment. This integrative schematic links three tissue modules—adipocytes, endothelial/microvascular cells, and macrophages—through lipid mediators, cytokines, and ARF6‐associated trafficking processes. In the adipocyte module, receptor signalling and lipolysis contribute to free fatty acid (FFA) release. In the endothelial module, cytokine‐related inflammatory input is depicted as acting on the cellular barrier/junctional compartment, where ARF6 is positioned at the membrane‐associated intracellular side to indicate its role in trafficking‐dependent regulation of barrier properties and insulin delivery, rather than as an extracellular factor. In the macrophage module, ARF6‐associated receptor trafficking is linked to inflammatory persistence, including NF‐κB‐associated signalling and cytokine output. The central closed‐loop hub summarises the proposed feed‐forward interaction among receptor trafficking, inflammatory amplification, and insulin resistance. Arrows represent conceptual directions of intercellular influence and signalling coupling rather than direct physical binding events or exact spatial localisation.

Obesity‐associated metabolic tissues are characterised by chronic low‐grade inflammation. This state is driven by macrophage infiltration and persistent inflammatory receptor signalling.^19^ In this context, ARF6 may contribute to maintenance or amplification of the local inflammatory microenvironment (Figure 5).^20^ ARF6 is considered relevant in this inflammatory context because it lies at the interface of receptor trafficking, membrane remodelling, and immune‐cell migratory behaviour, thereby potentially influencing both the persistence of inflammatory signalling and the spatial expansion of macrophage‐driven tissue inflammation. Figure 5 integrates adipocyte, endothelial, and macrophage compartments into a single inflammatory‐metabolic framework organised around ARF6‐associated receptor trafficking. This schematic is intended to illustrate how ARF6‐dependent trafficking processes may participate in a feed‐forward network linking free fatty acid release, cytokine signalling, endothelial dysfunction, and macrophage infiltration. In this model, ARF6 refers to intracellular membrane‐trafficking control within each cell type. The arrows between compartments indicate conceptual inflammatory coupling rather than direct extracellular actions of ARF6 itself. This framework is also consistent with the possibility that ARF6‐dependent receptor turnover helps sustain downstream inflammatory signalling, including NF‐κB‐associated pathways.

### 5.2 ARF6‐dependent receptor recycling and macrophage infiltration

ARF6 may influence the turnover of inflammatory receptors by promoting their endosomal recycling back to the cell surface rather than their lysosomal degradation. In this way, ARF6 could prolong cellular responsiveness to free fatty acids and cytokines and sustain inflammatory signalling.^21^ ARF6 may also contribute to macrophage infiltration into adipose tissue. Local actin cytoskeletal remodelling is required for macrophage chemotaxis and migration, and ARF6 appears to participate in this process.^22^ Consistent with this view, macrophage‐specific deletion of Arf6 has been reported to reduce local inflammatory responses and improve insulin sensitivity in experimental models.^23^ Taken together, these findings suggest that ARF6 in immune cells may affect both macrophage recruitment and the persistence of inflammatory signalling. This possibility provides part of the rationale for the translational strategies discussed later in this review. Those approaches are framed less as broad anti‐inflammatory suppression and more as selective targeting of macrophage‐enriched compartments in metabolically inflamed tissues. Recent in vivo evidence further supports the metabolic relevance of this pathway. Endothelial‐specific reduction of Arf6 has been shown to impair insulin‐mediated vasodilation and skeletal muscle blood flow, changes that were associated with systemic insulin resistance.^24^ This observation suggests that the metabolic effects of ARF6 may extend beyond macrophages alone and may involve coordinated changes across multiple cell types.

## 6 THE TRANSLATIONAL IMPERATIVE: FROM SYSTEMIC TOXICITY TO TARGETED NANODELIVERY

### 6.1 Why direct systemic modulation of ARF6 is translationally problematic

**FIGURE 6:**
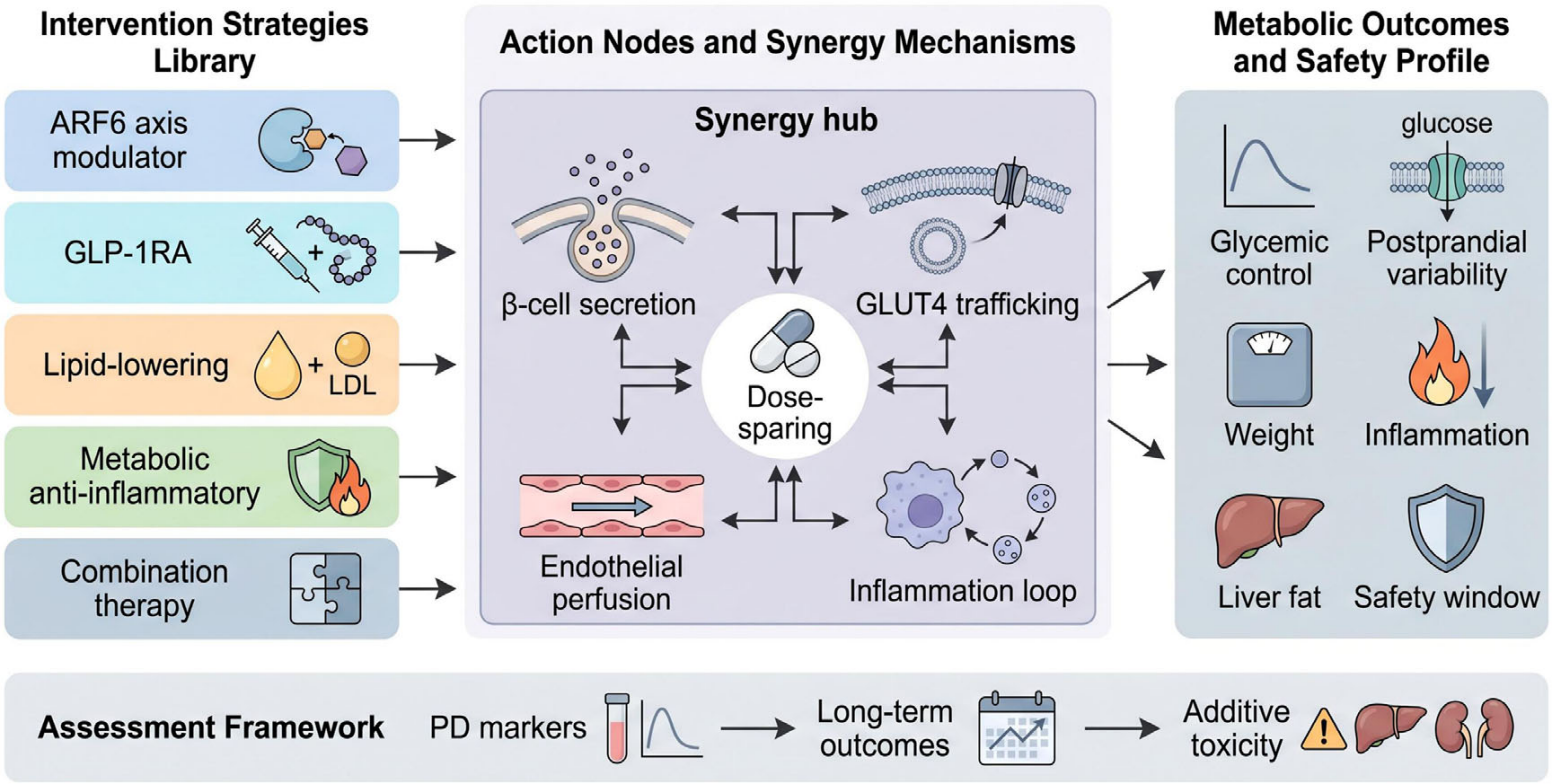
Integrated therapeutic framework for ADP‐ribosylation factor 6 (ARF6)‐directed intervention in metabolic disease. The left panel summarises candidate intervention classes, including ARF6‐axis modulators, GLP‐1 receptor agonists, lipid‐lowering agents, metabolic anti‐inflammatory approaches, and combination therapy. The central synergy hub depicts four functional action nodes through which these interventions may converge: β‐cell secretion, glucose transporter type 4 (GLUT4) trafficking, endothelial perfusion, and inflammatory loop control, with dose‐sparing shown as a potential integrative benefit of mechanistically complementary interventions. The right panel outlines representative translational outcomes, including glycemic control, postprandial variability, body weight, inflammation, liver fat, and safety window. The lower assessment strip emphasises the need to connect pharmacodynamic markers to long‐term outcomes and additive toxicity during translational evaluation.

The translational concepts discussed in this section are summarised in Figure 6. Rather than presenting ARF6‐directed intervention as a single‐agent approach, the figure outlines a modular therapeutic framework. It links different intervention classes to four functional nodes: β‐cell secretion, GLUT4 trafficking, endothelial perfusion, and inflammatory loop control. It also highlights dose reduction as a possible advantage of mechanistically complementary combinations. In addition, the figure summarises representative metabolic and safety outcomes that may be informative during translation. Pharmacological inhibition of ARF6 or its upstream guanine nucleotide exchange factors, including cytohesin‐targeting compounds such as SecinH3, may interrupt disease‐relevant membrane‐trafficking processes.^8^ However, direct systemic modulation of this pathway may also carry substantial safety concerns (Figure 6). ARF6 contributes to VE‐cadherin turnover and actin dynamics in endothelial cells. As a result, systemic inhibition could impair vascular barrier integrity and increase permeability risk.^25^, ^26^ A further complication may arise in the context of nucleic acid therapeutics such as small interfering RNA (siRNA) and antisense oligonucleotides (ASOs). Uptake of some nanocarriers depends on ARF6‐associated endocytic pathways. Systemic suppression of ARF6 could interfere with cellular entry of the therapeutic carriers themselves.^27^ This issue suggests that delivery strategy may be as important as target selection in efforts to translate ARF6‐directed interventions.

### 6.2 Tissue‐targeted and microenvironment‐responsive nanodelivery strategies

**FIGURE 7:**
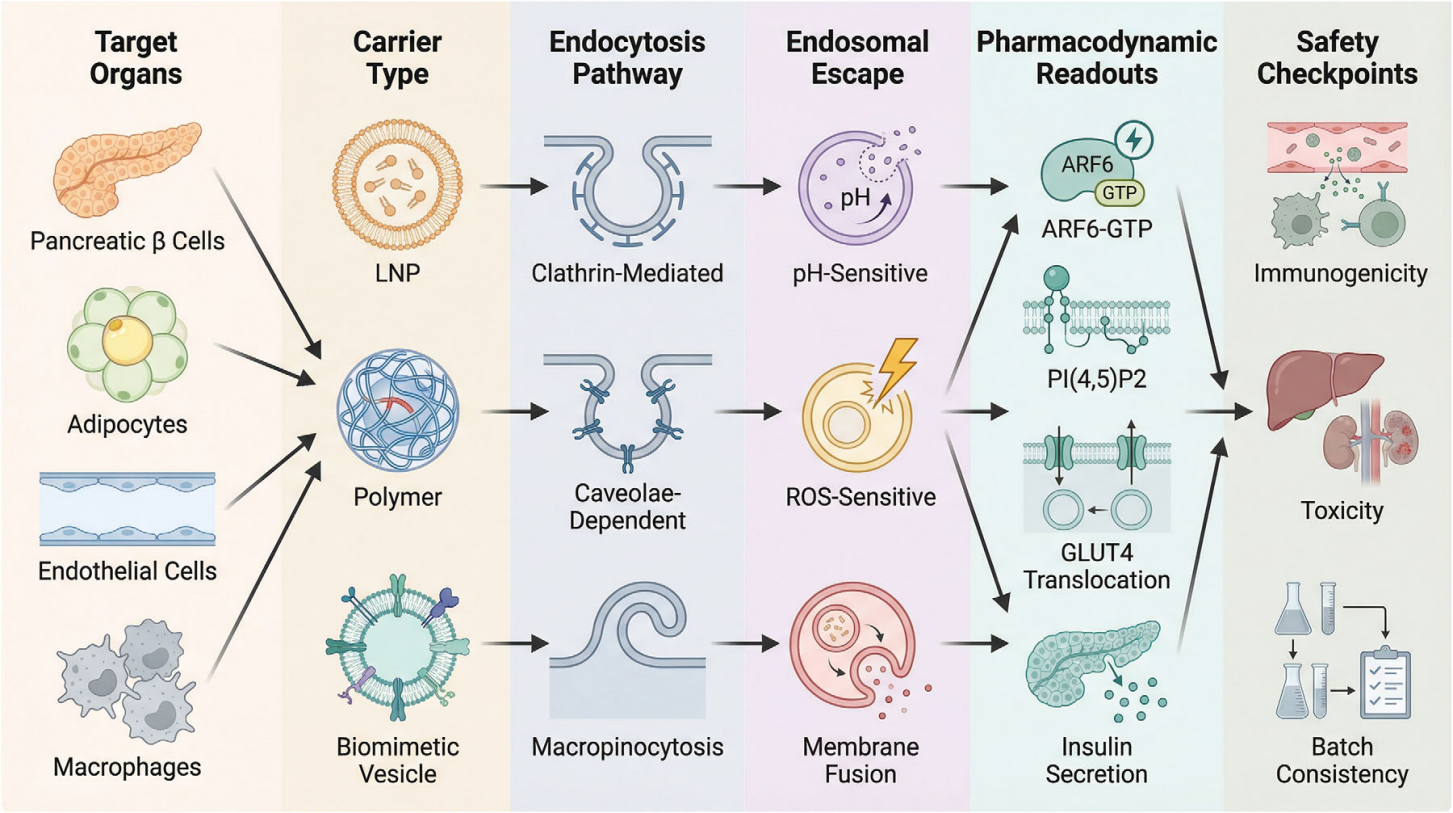
End‐to‐end nanodelivery design framework for targeting the ADP‐ribosylation factor 6 (ARF6) axis. This schematic links six translational design levels: target organs/cell types (e.g. β cells, adipocytes, endothelial cells and macrophages), carrier platforms (e.g. LNPs, polymers and biomimetic vesicles), endocytic uptake routes, endosomal escape mechanisms, pharmacodynamic readouts, and safety checkpoints. The purpose of the figure is to emphasise that successful ARF6‐directed therapy depends on coordination between mechanism and delivery, particularly because ARF6 itself influences endocytosis and membrane trafficking. Arrows indicate design progression from target selection to translational evaluation.

Figure 7 summarises the general design framework for nanodelivery systems aimed at the ARF6 axis. It links target tissues, carrier classes, endocytic entry routes, endosomal escape strategies, pharmacodynamic readouts, and safety checkpoints. In this framework, delivery design may strongly influence efficacy, tissue selectivity, and off‐target risk. Tissue‐targeted nanodelivery may offer a practical way to separate potential metabolic benefits from the risks associated with systemic exposure and broad endocytic interference (Figure 7).^28^

For pancreatic β‐cells, one possible strategy is the use of nanoparticles conjugated to Exendin‐4 analogues. Because Glucagon‐like peptide‐1 (GLP‐1) receptors are highly expressed in β‐cells, this approach may improve local delivery while limiting vascular exposure.^29^ In macrophages, phosphatidylserine‐mimicking or mannose‐modified carriers could help enrich ARF6 inhibitors in adipose tissue‐resident inflammatory cells, with the aim of reducing pathogenic receptor recycling and inflammatory persistence.^30^ For adipose tissue, biomimetic systems incorporating integrin‐targeting peptides may improve nanoparticle accumulation within poorly perfused adipose depots and thereby support local correction of trafficking defects relevant to GLUT4 handling.^31^ In addition, microenvironment‐responsive carriers, such as matrix metalloproteinase (MMP)‐cleavable systems, may remain relatively inactive in the circulation and release ARF6 modulators more selectively within inflamed lesions.^32^ Recent reviews of extracellular vesicle (EV)‐based and other biomimetic delivery systems further emphasise that source‐dependent targeting behaviour, EV‐cell interaction mechanisms, and manufacturing constraints are all likely to shape the translational feasibility of next‐generation nanocarriers.^33^ A simplified comparison of nanodelivery platforms that may be relevant to ARF6‐directed intervention is provided in Table 1.

**TABLE 1:** Simplified comparison of nanodelivery platforms potentially relevant to ARF6‐directed intervention.

Delivery platform	Main strengths	Main limitations	Potential relevance to ARF6‐directed therapy
Lipid nanoparticles (LNPs)^28^	Mature commercial platform; favourable in vivo distribution; suitable for nucleic acid therapeutics; modifiable for extrahepatic targeting	Hepatic tropism; immune activation risk; temperature/storage sensitivity	Potentially suitable for siRNA/ASO‐based ARF6‐axis modulation, especially if tissue distribution can be shifted beyond the liver
Polymeric nanoparticles (PLGA/PEI/PEGylated)^34^	Tunable structure; sustained release; co‐loading of small molecules and nucleic acids	Limited endosomal escape; cytotoxicity of some cationic polymers	May support tissue‐tailored delivery of ARF6 modulators where controlled release and formulation flexibility are priorities
Cell membrane–mimetic nanocarriers (RBC membrane, macrophage membrane)^35^	Immune evasion; prolonged circulation; active targeting (e.g. inflammatory foci)	Complex preparation; immunological uncertainty from heterologous sources	Potentially attractive for macrophage‐ or inflammation‐directed ARF6 targeting in metabolically inflamed tissues
Exosomes/natural vesicles^36^	Native endocytic pathways; strong barrier‐crossing capacity; suitable for chronic dosing	Low yield; limited loading efficiency; challenging cargo control	May offer a biologically compatible route for chronic ARF6‐directed delivery, although translational standardisation remains challenging

## 7 CONCLUSION

In summary, the ARF6 signalling axis may function as a membrane‐trafficking and cytoskeletal regulatory node linking insulin secretion, peripheral glucose handling, and metabolic inflammation. Its therapeutic relevance lies less in acting as a universal master regulator and more in its position at the interface of secretion, transporter recycling, and inflammatory persistence in a context‐dependent manner. At the same time, this position also makes therapeutic targeting more difficult. Because ARF6 participates in essential membrane‐trafficking functions across multiple cell types, broad systemic modulation may disrupt normal homeostatic processes. Future progress will likely depend on moving away from nonspecific inhibition and toward tissue‐selective, stage‐appropriate, and mechanistically informed intervention. In this setting, combining ARF6‐directed strategies with tissue‐targeted or microenvironment‐responsive nanodelivery systems may offer a more feasible translational path to improve glucose homeostasis while limiting systemic toxicity. However, ARF6 should not be viewed as the only major regulatory axis in glucose metabolism. Glucose homeostasis is governed by multiple interacting pathways, including canonical insulin receptor‐PI3K‐Akt signalling, AMPK‐dependent energy sensing, Rab/SNARE‐mediated vesicle trafficking, and inflammatory programs involving NF‐κB and related networks. Accordingly, this review positions ARF6 as one potentially important mechanistic and therapeutic entry point within a broader regulatory landscape, rather than as an exclusive master regulator.

## AUTHOR CONTRIBUTIONS

Yangyang Wang conceived and designed the study. Yangyang Wang, Zhiming Xiu, Huiyan Wang and Yan He performed the literature investigation and data analysis. Yangyang Wang wrote the manuscript. All authors reviewed and approved the final version of the manuscript.

## CONFLICT OF INTEREST STATEMENT

The authors declare no conflict of interest.

## ETHICS STATEMENT

Not applicable.

## Data Availability

No new data were generated or analysed in this study. All information discussed in this review is derived from previously published studies cited in the article.
